# From Kidney to Brain: An Uncommon Severe Relapse of Myeloperoxidase Anti-Neutrophil Cytoplasmic Antibody (MPO-ANCA) Vasculitis

**DOI:** 10.7759/cureus.14205

**Published:** 2021-03-31

**Authors:** Tiago Araújo, Rúben Maia, João Massano, Luis Mendonça, Joana Guimarães

**Affiliations:** 1 Department of Internal Medicine, Unidade Local de Saúde do Litoral Alentejano, Santiago do Cacém, PRT; 2 Department of Neurology, Centro Hospitalar Universitário de São João, Porto, PRT; 3 Department of Neuroradiology, Centro Hospitalar Universitário de São João, Porto, PRT; 4 Department of Clinical Neurosciences and Mental Health, Faculdade de Medicina da Universidade do Porto, Porto, PRT; 5 Department of Nephrology, Centro Hospitalar Universitário de São João, Porto, PRT

**Keywords:** anca-associated vasculitis, mpo-anca, central nervous system, ischemic lesions, rituximab

## Abstract

Anti-neutrophil cytoplasmic antibody (ANCA)-associated vasculitis (AAV) is a group of rare autoimmune diseases that affect medium and small blood vessels, with uncommon, variable central nervous system (CNS) involvement. It poses diagnosis challenges due to the limited accuracy of conventional imaging and vast differential diagnosis.

We describe the case of a 76-year-old man with a previously diagnosed myeloperoxidase (MPO)-positive AAV with exclusive renal involvement. The patient presented to our emergency department (ED) with sudden-onset weakness of the right side of the body, difficulty speaking, fever, and a history of progressive cognitive impairment in the previous three months (loss of memory, time and space disorientation, acalculia). Brain imaging showed multiple acute and subacute ischemic lesions, suggesting CNS vasculitic involvement. The patient was treated with methylprednisolone pulses, followed by rituximab, with motor and cognitive improvement.

Timely diagnosis and adequate treatment of AAV as a cause for new-onset neurological symptoms are crucial to improve outcomes. Otherwise, a higher risk of relapse remains, and extensive neurological deficits may become permanent. Evidence regarding the best treatment options in these patients is scarce and case reports provide further data on this topic.

## Introduction

Anti-neutrophil cytoplasmic antibody (ANCA)-associated vasculitis (AAV) have an estimated prevalence of 4.6-18.4 cases per 100,000 individuals [1]. The central nervous system (CNS) manifestations of AAV occur in less than 15% of patients and are variable, ranging from headache to spinal cord symptoms, but also intracranial hemorrhage or ischemic infarctions [2]. The diagnosis is reached by careful consideration of clinical findings in conjunction with serology, imaging, and even pathological data [2]. Reports of AAV presenting as stroke are rare and its treatment is based on high-dose corticosteroids, immunosuppressive drugs, and sometimes plasmapheresis [3]. Relapses are not unusual in AAV and the associated morbidity is high. The persistence of ANCA antibodies after induction therapy or an increase in its titers are known major risk factors for relapse [4]. Severe relapses with organ- or life-threatening conditions like diffuse alveolar hemorrhage, rapidly progressing renal failure, peripheral or central nervous system involvement, gastrointestinal ischemia, or sight-threatening ocular disease (retinitis, retinal vasculitis, scleritis, orbital pseudotumor), must be treated as a new-onset disease [2].

## Case presentation

A 76-year-old man with a personal history of hypertension, type two diabetes mellitus, hypercholesterolemia, chronic kidney disease secondary to MPO positive AAV with exclusive renal involvement, and a recent history of minor ischemic stroke of unknown etiology (National Institute of Health Stroke Score of 3 at the time of discharge) presented to our emergency department (ED) with sudden-onset weakness in the right side of the body, difficulty speaking, fever (isolated spike of 38.2°C) and a history of progressive cognitive impairment in the previous three months. His autoimmune condition initially presented five years ago as pauci-immune crescentic glomerulonephritis, confirmed by renal biopsy. Induction immunosuppression included pulses of methylprednisolone and intravenous cyclophosphamide (cumulative dose of 7250 mg), followed by maintenance therapy with prednisolone (suspended in 2016) and azathioprine (100 mg daily). Although he remained in clinical remission, ANCA and erythrocyte sedimentation rate (ESR) levels were persistently high even during maintenance-remission therapy, at 144 U/ml (normal range <20 U/ml) and 66 mm/hr (normal range 0-20 mm/hr), respectively. 

On physical examination, the patient was able to follow simple commands (close eyes, grasp hand), however, presented with time and place disorientation, marked verbal fluency impairment, anomic aphasia, right-sided hemiparesis (grade 4 on the Medical Research Council Manual Muscle Testing Scale) with no involvement of the face and moderate global bradykinesia. He also presented diplopia on the left lateral gaze and skew deviation of the right eye due to a previous stroke. He was febrile (auricular temperature 38°C). A brain non-enhanced computed tomography (CT) was performed and revealed a subacute right thalamic infarct and a left frontal lacune not seen in the CT scan performed three months before. CT angiography of the head and neck excluded large vessel occlusions. Laboratory data showed normocytic and normochromic anemia with hemoglobin of 11.9 g/dL, white blood cell count of 7.38 x10^9^/L, platelet count of 158 x10^9^/L, stable renal function (urea of 66 mg/dl and creatinine of 1.52 mg/dl), normal urinalysis, slightly elevated C-reactive protein of 6.2 mg/L (normal range <3.0 mg/L), ANCA levels of 144 U/ml, and ESR of 65 mm/hr. Electrocardiogram showed a first-degree atrioventricular block and the chest X-ray was within normal limits. He was admitted for treatment of acute stroke and for etiologic study. At this point, a lumbar was not performed due to low suspicion of CNS infection.

**Figure 1:**
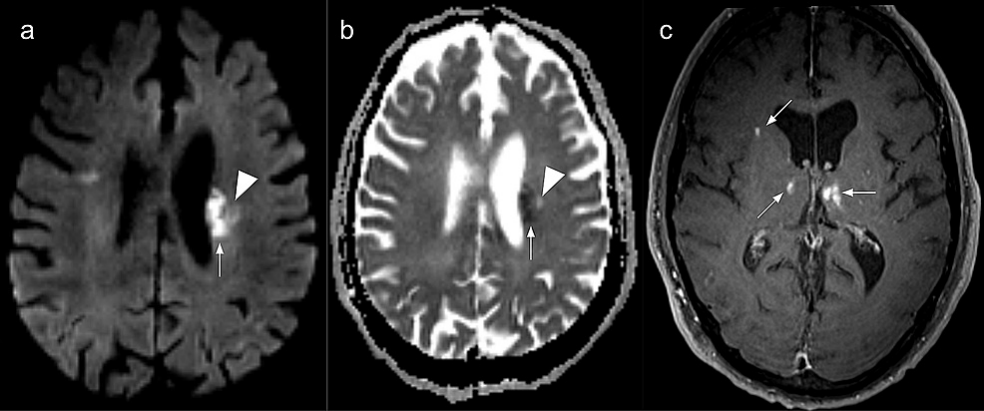
Brain magnetic resonance imaging Multiple and bilateral ischemic lesions in various evolution stages. (a) Axial diffusion-weighted image and (b)  apparent diffusion coefficient (ADC) map showing an acute periventricular ischemic lesion (arrows). A chronic lesion is also seen as a facilitated diffusion area (arrowheads); (c) Axial contrast-enhanced T1-weighted image depicting enhancing subacute ischemic in both thalami and the right corona radiata.

During the first twenty-four hours after admission, the patient experienced significant worsening of the neurological deficits, with right hemiplegia, ipsilateral facial central palsy, and moderate dysarthria. Consequently, a brain magnetic resonance imaging (MRI) was performed and revealed an acute left superior striatocapsular infarct extending to the ipsilateral corona radiata and several other bilateral subacute ischemic infarcts (Figure 1).

**Figure 2:**
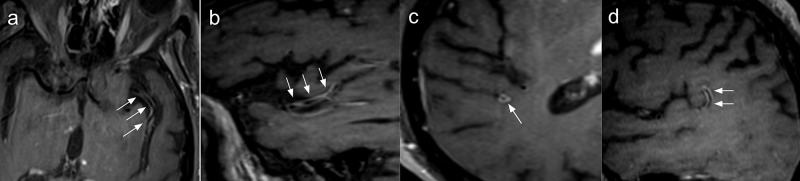
Brain magnetic resonance angiography with vessel-wall imaging Vessel wall imaging study with axial (a, c) and sagittal (b,d) reformats showing predominantly concentric thickening and enhancement of the left (arrows in a and b) and right (arrows in c and d) middle cerebral arteries.

Multiple vascular irregularities in both anterior and posterior circulation arteries were found on the magnetic resonance angiography and, most importantly, vessel-wall imaging (VWI) identified segmental concentric enhancement of M2 and M3 branches of the middle cerebral arteries, the right pericallosal artery, the right vertebral artery, and the proximal segment of the basilar artery, highly suggestive of vasculitis-associated inflammatory process (Figure 2).

Although the patient presented only two minor clinical criteria of the Duke criteria for the diagnosis of infective endocarditis (fever and arterial emboli), since he experienced recurrent fever, a transthoracic echocardiogram was performed, showing normal valvular anatomy; lumbar puncture excluded CNS infection, revealing elevated levels of proteins (0.97 g/L, normal range 0.15-0.45 g/L) with normal cytology, glucose, and adenosine deaminase levels in the cerebrospinal fluid. Venereal disease research laboratory (VDRL), bacteriologic, mycologic, and mycobacteriologic CSF tests were negative, as were polymerase chain reaction assays for the varicella-zoster virus, cytomegalovirus, Epstein-Barr virus, herpes simplex viruses 1 and 2, *Mycobacterium tuberculosis*, *Listeria, *and *Toxoplasma gondii*. Blood cultures were also negative.

After confirmation of negative screening for latent tuberculosis and negative serology for hepatitis B, C, and A viruses and human immunodeficiency virus, the patient was started on pulse steroid therapy consisting of three doses of 500 mg of methylprednisolone, followed by oral corticosteroid therapy with prednisone (1 mg/kg), and rituximab 500 mg (two consecutive weekly doses). In spite of that, after the first dose of rituximab, the patient showed only minor dysarthria and right lower limb muscle power improvements (grade 2/5), continued to experience time and place disorientation, and right upper limb monoplegia. He was discharged on the 29th day post-admission. He was re-examined on the 43rd day after the initial event, when he returned for his second dose of rituximab, presenting further signs of recovery: he was able to walk with the aid of another person, his verbal fluency improved, and he presented only sporadic periods of time and place disorientation, especially at the end of the day.

## Discussion

After the 2012 International Chapel Hill Consensus Conference, systemic vasculitis are classified according to the size of the involved blood vessel (small, medium, large, variable) [5]. In AAV, deposition of immune complexes causes inflammation and destruction of small- and medium-sized blood vessels. This condition is divided mainly into four clinicopathological variants: microscopic polyangiitis (MPA), granulomatosis with polyangiitis (GPA), eosinophilic granulomatosis with polyangiitis (EGPA), and single-organ involvement [5]. However, there is growing concern about how well these clinical phenotypes can be differentiated since many times they present overlapping clinical manifestations, primarily between MPA and GPA. In fact, it has been proposed that AAV classification should be based on the type of ANCA: anti-myeloperoxidase (MPO) or anti-proteinase 3 (PR3) [6]. Both relapse rates and clinical outcomes have a better correlation with ANCA specificity than with clinical phenotype [5]. MPO-positive AAV are associated with fewer relapses, but increased risk of initial treatment failure and a global worse prognosis [6].

Single-organ vasculitis is a rare form of vasculitis where blood vessel inflammation is confined to a specific organ [7]. In order to confirm this diagnosis, the existence of systemic disease must be excluded. It is also important to keep in mind that long-term follow-up is needed because some of these patients may later present with other organ involvement, demanding reclassification [5]. Renal-limited vasculitis usually presents with necrotizing crescentic glomerulonephritis and the majority of patients are ANCA positive, with 70-80% being MPO-ANCA [8,9].

Although rare, involvement of the CNS is serious and linked to poor prognosis. The range of CNS manifestations is wide and depends on the affected territory [10]. When brain parenchyma is affected, cerebrovascular events may be observed but also posterior reversible encephalopathy syndrome, isolated parenchymal mass lesions, or cognitive dysfunction. If brain meninges are affected, hypertrophic pachymeningitis may occur. Moreover, when the pituitary gland or stalk are involved, a variety of endocrine disorders can ensue: diabetes insipidus, hypogonadism, hypothyroidism, adrenocorticotropic hormone deficiency, hyperprolactinemia, and also loss of vision caused by optic chiasm compression. Finally, the spinal cord can similarly be affected, either by necrotizing inflammation of the spinal vasculature, compression of the spinal cord, or development of primary spinal granulomas [2].

A retrospective study conducted by Ma et al. studied 29 patients with AAV and CNS involvement in order to understand clinical and radiological manifestations and also their outcomes. They concluded that the main manifestation of CNS involvement occurred in the form of cerebral ischemic lesions, and these patients had a significantly more active AAV when compared with the ones without CNS involvement [11].

The occurrence of CNS manifestations in patients with uncontrolled AAV should prompt consideration that this could be the underlying cause. Still, the more common causes of neurological events must always be excluded (other vasculopathies, thrombotic conditions or embolic conditions, infections, malignancies). On the other hand, if the disease is controlled, therapy-related complications or disease relapse should be considered [10].

Confirmation of CNS involvement by AAV is made by combining neurological symptoms, ANCA titers, imaging techniques, CSF analysis, and, sometimes, even cerebral biopsy [3]. Brain MRI should be performed in every patient, and it may show various lesion patterns, including ischemic lesions of various ages affecting grey and white matter, pachymeningitis, microbleeds, tumor-like enhancing granulomatous lesions, and multiple arterial segmental and focal stenosis, occlusions, and fusiform dilations [10,12]. CSF fluid analysis usually shows pleocytosis and elevated protein levels, but its importance is also key in order to exclude alternative diagnoses (infection, malignancies, other inflammatory diseases) [12,13].

ANCA testing is also essential, and although not diagnostic when positive, it is very suggestive of AAV. However, a negative test does not exclude the diagnosis of AAV. In terms of the utility of ANCA titers for disease monitoring, prediction of relapse, and prognosis, there is still much controversy [14]. According to Lionaki et al., relapses seem more frequent in PR3-positive AAV versus MPO-positive [15]. However, a 2012 meta-analysis published by Tomasson et al. showed that a rise or persistence in ANCA levels during clinical remission had only a modest predictive value for relapse [16]. Likewise, the European League Against Rheumatism (EULAR) 2016 recommendations for the management of AAV clearly state that structured clinical assessment rather than ANCA testing should guide decisions on changes in treatment for AAV [17].

Treatment of AAV is divided into two phases - remission-induction and remission-maintenance - and it includes steroid and immunosuppressive therapy. Induction treatment usually consists of the combination of high-dose glucocorticoids and cyclophosphamide or rituximab [18]. If remission is attained, treatment should be switched to low-dose glucocorticoid and an oral immunosuppressive agent. Alternatively, recent expert consensus guidelines recommend the long-term use of rituximab for maintenance therapy in AAV [19]. If non-severe relapse occurs, a change in the immunosuppressive regimen must be considered. On the contrary, when relapse is deemed severe, induction treatment should be repeated. Furthermore, in patients presenting with serum creatinine >5.7 mg/dl due to rapidly progressive glomerulonephritis in the setting of new or relapsing disease, plasma exchange therapy should be considered [17]. Since CNS involvement by AAV is considered an organ-threatening event, when it occurs in a patient with previously diagnosed AAV - like the case we here present - that is the correct option [17]. Several factors may influence the choice between cyclophosphamide and rituximab for induction therapy: the high cost of rituximab; the desire of the patient to preserve reproductive potential that may be hindered by cyclophosphamide since it is associated with a decrease in ovarian reserve, ovarian failure, and male infertility; the risk of alopecia that may be relevant for some patients; and the previous cumulative dose of cyclophosphamide in cases where it is needed to repeat induction therapy due to severe relapse, as occurred with our patient.

In the present case, clinical remission was attained, with no signs of disease for many years, but the patient showed persistent ANCA levels and high ESR. Given that there was no change from baseline ANCA/ESR values, and the patient had several risk factors for cerebrovascular disease at the time he presented with new-onset neurological deficits, alternative causes of stroke were initially considered. However, neuroimaging was quite useful as it showed clear signs of vasculitic involvement of the brain and cerebral arteries, particularly aided by VWI sequences. Induction treatment was initiated and the patient improved with regard to the motor and cognitive deficits.

## Conclusions

CNS involvement by ANCA-associated vasculitis is a rare condition that can manifest itself in many different forms, and thus has a wide differential diagnosis. Clinical suspicion aided by ANCA titers and other laboratory findings, imaging techniques (mainly brain MRI), and cerebrospinal fluid analysis are key in the diagnosis. Timely diagnosis and treatment are vital in the management of these patients to obtain remission and improve outcomes.
